# Brilliant whiteness in shrimp from ultra-thin layers of birefringent nanospheres

**DOI:** 10.1038/s41566-023-01182-4

**Published:** 2023-04-24

**Authors:** Tali Lemcoff, Lotem Alus, Johannes S. Haataja, Avital Wagner, Gan Zhang, Mariela J. Pavan, Venkata Jayasurya Yallapragada, Silvia Vignolini, Dan Oron, Lukas Schertel, Benjamin A. Palmer

**Affiliations:** 1 Department of Chemistry, Ben-Gurion University of the Negev, Beer-Sheva, Israel; 2 Department of Molecular Chemistry and Materials Science, Weizmann Institute of Science, Rehovot, Israel; 3 Department of Chemical and Structural Biology, Weizmann Institute of Science, Rehovot, Israel; 4 Yusuf Hamied Department of Chemistry, University of Cambridge, Cambridge, UK; 5 Department of Applied Physics, Aalto University School of Science, Espoo, Finland; 6 Ilse Katz Institute for Nanoscale Science & Technology, Ben-Gurion University of the Negev, Beer-Sheva, Israel; 7 Department of Physics, Indian Institute of Technology Kanpur, Kanpur, Uttar Pradesh India; 8 Department of Physics, University of Fribourg, Fribourg, Switzerland; 9 Present Address: College of Chemistry and Chemical Engineering, Lanzhou University, Lanzhou, China

**Keywords:** Biophotonics, Optical materials, Photonic crystals, Biophysics, Green photonics

## Abstract

A fundamental question regarding light scattering is how whiteness, generated from multiple scattering, can be obtained from thin layers of materials. This challenge arises from the phenomenon of optical crowding, whereby, for scatterers packed with filling fractions higher than ~30%, reflectance is drastically reduced due to near-field coupling between the scatterers. Here we show that the extreme birefringence of isoxanthopterin nanospheres overcomes optical crowding effects, enabling multiple scattering and brilliant whiteness from ultra-thin chromatophore cells in shrimp. Strikingly, numerical simulations reveal that birefringence, originating from the spherulitic arrangement of isoxanthopterin molecules, enables intense broadband scattering almost up to the maximal packing for random spheres. This reduces the thickness of material required to produce brilliant whiteness, resulting in a photonic system that is more efficient than other biogenic or biomimetic white materials which operate in the lower refractive index medium of air. These results highlight the importance of birefringence as a structural variable to enhance the performance of such materials and could contribute to the design of biologically inspired replacements for artificial scatterers like titanium dioxide.

## Abstract (web summary)

The strong birefringence of liquid crystalline nanospheres in the body of the Pacific cleaner shrimp enables brilliant whiteness by overcoming undesirable optical crowding effects.

## Main

White colours are produced by diffuse light propagation in disordered media^1^. To generate whiteness, photons of all wavelengths must be scattered multiple times and lose their directional information to produce broadband, angle-independent reflectance. Although this is easily achieved with thick samples^2^, it is difficult to obtain with thin layers of material^3^. Recent studies have explored how ultra-thin photonic structures can be tuned to produce efficient scattering^1,3–11^, which is relevant to sensors, solar cells, displays and enhanced absorbers^12–15^. This is achieved by optimizing the combination of single-particle scattering (the form factor—particle refractive index, shape and size) and particle ensemble scattering (the structure factor—packing density and layer thickness). To produce artificial white materials, industry has relied on use of the high-refractive-index inorganic materials TiO_2_ (*n* ≈ 2.6), ZnS (*n* ≈ 2.4) and ZnO (*n* ≈ 2.0)^16–18^. However, due to health concerns, the most common whitening agent, TiO_2_, has recently been banned as a food additive by the European Union. In 2022, the European Medicines Agency also stressed the ‘critical importance’ of finding benign replacements for TiO_2_ in medicines^19^.

An obvious strategy for identifying alternative materials is to seek inspiration from biology. However, biological examples of ‘enhanced whiteness’ produced by ultra-thin scatterers are rare and, like the *Cyphochilus* beetle (5–10 μm thick)^20–25^ or the *Pieris rapae* butterfly (<5 μm thick)^26–28^, typically rely on a medium of air (*n* = 1) to generate sufficient refractive-index contrast. Achieving the same task in a solid film or in water, where the refractive indices of the medium are higher, poses a much bigger challenge.

In this Article we show how the brilliant white regions of the cleaner shrimp are generated by multiple scattering from thin films of birefringent particles. The impressive white colours, which provide effective signalling in an aqueous habitat, are produced by chromatophore cells containing dense arrays of high-refractive-index (*n* ≈ 2.0), birefringent (~30%) isoxanthopterin nanospheres. A particularly striking result is that extreme birefringence, which originates from the spherulitic arrangement of isoxanthopterin molecules, non-intuitively diminishes the unfavourable effects of optical crowding, which drastically reduces reflectance at high packing densities. Birefringence thus enables increased scattering, even compared with similar high-index isotropic nanospheres, and plays a crucial role in reducing the thickness of material required to produce high scattering in an aqueous medium. Our results provide a pathway towards new, artificial ultra-white, ultra-thin layers made from biodegradable organic nanospheres.

## Results

### Bright whiteness from ultra-thin chromatophores in shrimp

**Fig. 1:**
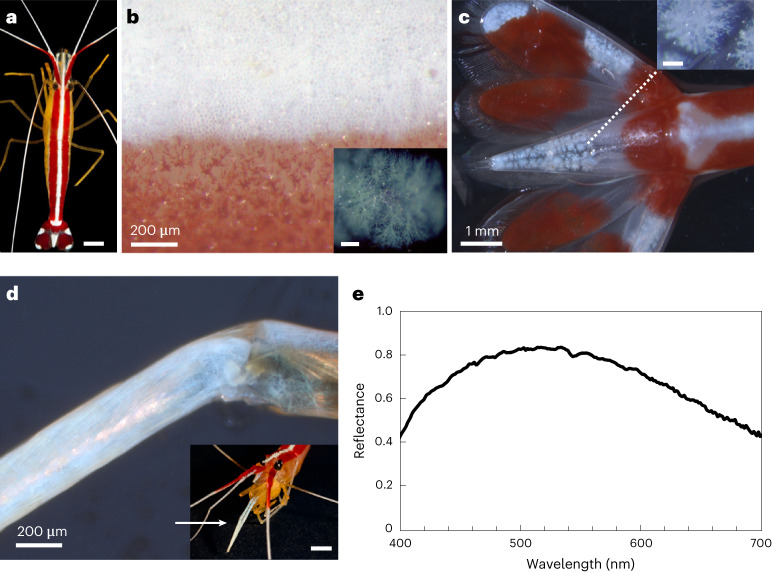
Brilliant white dermal chromatophore cells in the Pacific cleaner shrimp. **a**, The Pacific cleaner shrimp (*Lysmata amboinensis*). Scale bar, 0.5 cm. **b**, Optical micrograph of the white stripe on the carapace. Inset: the dendritic white chromatophores. Inset scale bar, 100 μm. **c**, Optical micrograph of white chromatophores on the tail. Inset: higher-magnification image of a white chromatophore. Inset scale bar, 100 μm. **d**, Optical micrograph of the maxilliped. Inset: the anatomical location of the maxillipeds. Inset scale bar, 0.5 cm. **e**, Average reflectance spectra of five measurements obtained from the white stripe using a ×40 water immersion objective (Supplementary Figs. 2–5). Credit: **a**, © Joel Sartore/Photo Ark.

The Pacific cleaner shrimp (*Lysmata amboinensis*) is a tropical shrimp that feeds on parasites and dead tissue on client fish^29^. The shrimp has a bright white stripe along its carapace (Fig. 1a,b), a symmetrical bright white pattern on its tail (Fig. 1c), white antennae (Fig. 1a) and two white maxillipeds (Fig. 1d). The white colour arises from a single layer of dendritic white chromatophore cells (Fig. 1b–d), whose degree of interdigitation varies across the white regions, with the cells of the dorsal stripe being the most densely interdigitated and those of the antennae the least dense (Supplementary Fig. 1). Microspectrophotometry reflectance measurements show that the dorsal stripe displays intense reflectance (50–80%) across the visible (Fig. 1e and Supplementary Figs. 2–5), resulting in a matte white appearance.

**Fig. 2:**
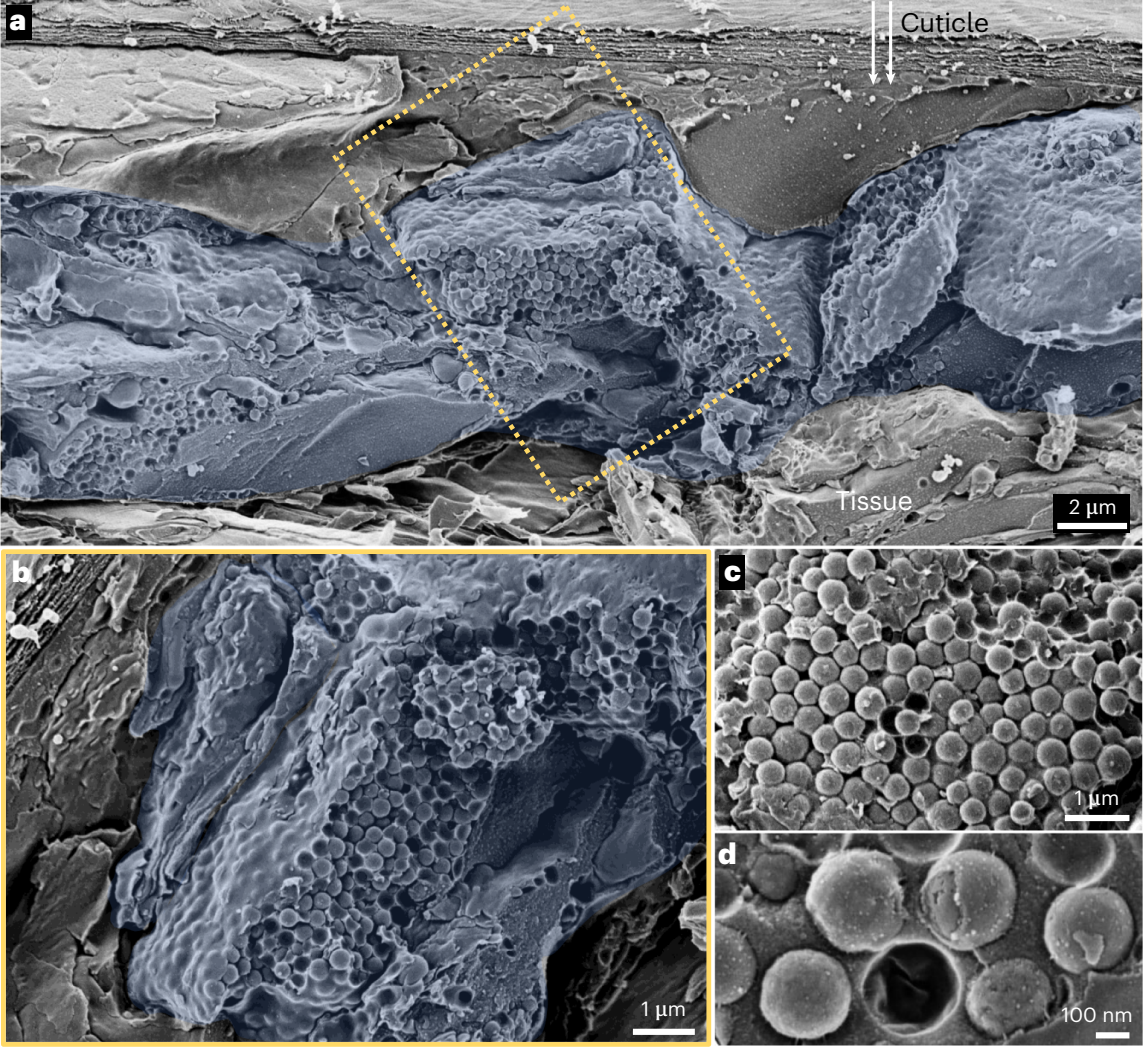
Cryo-SEM images of the white chromatophore cells. **a**, Low-magnification image of a cross-section through the white maxilliped, displaying the white cells (pseudo-coloured blue) underlying the transparent chitin cuticle. Arrows indicate the direction of incident light. **b**, Higher-magnification image of the region of interest (rotated) in **a**, showing a chromatophore cell and its component nanospheres. **c**, Region from the antenna showing the same dense particle packing. **d**, A cluster of particles located in the maxilliped.

To elucidate the ultrastructural properties of the white cells and the nature of the component optical material, we performed cryo-scanning electron microscopy (cryo-SEM) on cryo-immobilized, freeze-fractured tissues. Cryo-SEM images of the white maxillipeds (Fig. 2a,b) and antennae (Fig. 2c) show that the exceptionally thin (3–7 μm) chromatophores underlying the cuticle contain very densely packed nanosphere organelles (305 ± 31 nm in diameter; Fig. 2d and Supplementary Fig. 6). Similar particles were also extracted from the dorsal stripe (Supplementary Fig. 7). These observations are consistent with those of Elofsson et al.^30^, who showed that transmission electron microscopy (TEM) images of white chromatophores in sand shrimp (*Crangon crangon*) exhibit round, membrane-bound voids.

### Chemical and structural properties of the nanospheres

For intense whiteness to be produced by such a thin material in an aqueous medium (*n*_cytoplasm_ ≈ 1.33), the particles would have to possess a very high refractive index to generate sufficient index contrast. Moreover, the dense particle packings (~50–65%) observed here are usually not optimal for efficient whiteness for two reasons: first, high densities typically lead to short-range positional order and the emergence of colour^31^. Second, dense packings usually lead to loss of scattering efficiency due to near-field coupling^6,32^. Determining the chemical, structural and optical properties of individual particles is key to understanding the mechanisms used to optimize whiteness in this system and its deviation from ‘common’ design rules.

**Fig. 3:**
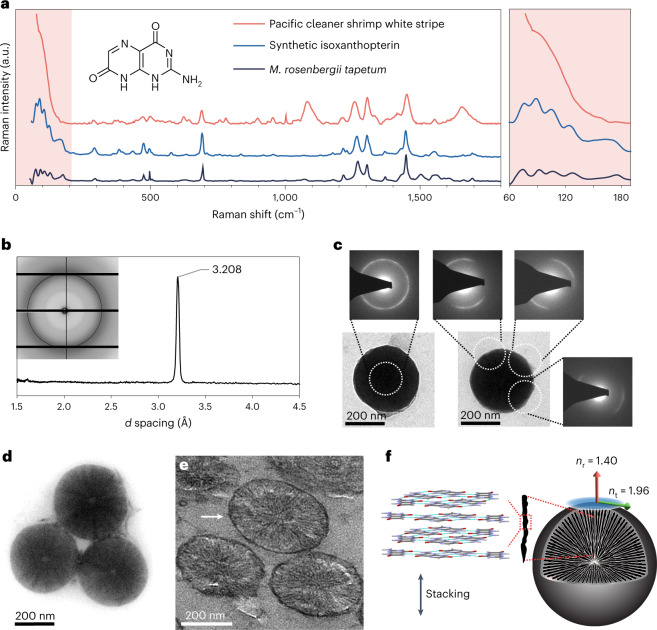
Chemical and structural properties of the reflective nanospheres. **a**, Raman spectra from the white stripe of the cleaner shrimp, overlaid with the spectra of crystalline biogenic and synthetic isoxanthopterin. Inset: molecular structure of isoxanthopterin. Right: low-frequency region of the cleaner shrimp spectrum. The broad peak at ~1,080 cm^−1^ corresponds to amorphous calcium carbonate from the cuticle^55^. The peak at ~1,600–1,700 cm^−1^ is probably an amide I band, and the sharp peak at ~1,000 cm^−1^ is a phenylalanine aromatic ring stretch, both associated with proteins^56^. There are several other peaks whose chemical origin is not yet identified. **b**, In situ WAXS diffraction pattern from the maxilliped (obtained by radial integration of the 2D scattering pattern; inset). **c**, TEM images and corresponding SAED of two particles with *d* spacing ~3.2 Å. The reflection angle changes when moving along the dihedral angles of the particle, indicating that the stacking axis projects radially away from the centre of the sphere. **d**, A TEM image of three extracted particles exposing the internal spoke-like structure. **e**, TEM micrograph of the nanospheres in an ultra-thin tissue section (~100 nm), showing the spoke-like, spherulitic structure. The white arrow denotes the nanosphere membrane. **f**, Schematic of a nanosphere showing the spherulitic arrangement of stacked isoxanthopterin molecules. Radial (*n*_r_) and tangential (*n*_t_) refractive index vectors on the nanosphere surface illustrate the birefringence produced from the spherulitic arrangement. Dark-blue trace in panel **a** adapted with permission from ref. ^37^, AAAS.

To elucidate the chemical identity of these particles, Raman spectra were collected from the dorsal stripe (Fig. 3a), which showed that the particles are mainly composed of the heterocyclic molecule isoxanthopterin. Thin layer chromatography showed that isoxanthopterin was present in all white regions of the shrimp (Supplementary Fig. 8). In its crystalline form, isoxanthopterin has been shown to be the optical material in the tapetum lucidum reflector of decapod crustacean eyes^33^. Isoxanthopterin crystals are constructed from stacked, planar hydrogen-bonded molecular layers and possess a high refractive index (density functional theory (DFT)-calculated mean value of *n* = 1.96^33^) parallel to the hydrogen-bonded (200) plane (Supplementary Fig. 9). In contrast, the out-of-plane refractive index (parallel to the stacking direction) is calculated to be 1.40^33^. In the tapetum reflector, the isoxanthopterin crystals are assembled into nanospheres constructed from concentric lamellae of nanoscopic single crystals, in which the (200) crystal faces project radially from the surface of the sphere. This spherulitic arrangement orients the high in-plane refractive index tangentially to the particle surface, which substantially enhances the scattering efficiency of the particles^34–36^. In contrast to crystalline isoxanthopterin^33,37^, the Raman spectrum from the cleaner shrimp does not exhibit sharp, low-frequency peaks corresponding to lattice modes (Fig. 3a, inset), indicating that, here, the isoxanthopterin molecules do not exhibit three-dimensional (3D) periodicity.

To investigate the structural properties of the particles, in situ synchrotron μ*-*spot wide-angle X-ray scattering (WAXS; Fig. 3b and Supplementary Figs. 10 and 11) and electron-diffraction investigations were performed (Fig. 3c and Supplementary Fig. 12). WAXS patterns from the dorsal stripe and maxilliped exhibit a single sharp Bragg peak consistent with a partially crystalline material (Fig. 3b and Supplementary Fig. 10). The peak has a *d* spacing of 3.2 Å, which corresponds to the (200) reflection of isoxanthopterin (equivalent to the stacking distance)^33,34^. Similarly, electron-diffraction patterns of whole particles exhibit a single diffraction ring with a spacing of ~3.25 Å. Selected area electron diffraction (SAED) performed on different locations along the particle edge (Fig. 3c) exhibit diffraction arcs, the azimuthal angle of which follows the dihedral angle of the sphere. These data show that the nanospheres are composed of 1D ordered, stacked assemblies of isoxanthopterin that project radially outwards from the centre of the sphere (that is, a spherulite). The azimuthal spread of the scattering shows that the isoxanthopterin assemblies have a range of orientations. TEM (Fig. 3d,e) images reveal that the membrane-bound particles contain high-contrast spokes (<10 nm thick) that project from the core of the nanosphere like the spokes of a wheel (Fig. 3f). These spokes probably correspond to the stacked isoxanthopterin assemblies (Fig. 3c–f and Supplementary Fig. 12). The molecular orientation of the nanospheres is thus identical to that of crystalline isoxanthopterin^33,38^, but, in this case, 3D periodicity is absent. The presence of Raman peaks associated with proteins suggests that the nanospheres may have a more complex composition that could relate to their stabilization in a semicrystalline state or to the spherulitic arrangement of component isoxanthopterin molecules (Fig. 3a). WAXS patterns from white regions in *Neocaridina davidi* and *Caridina breviata* shrimp and electron diffraction of extracted nanospheres from *Lysmata debelius* exhibit the same diffraction properties (Supplementary Figs. 11 and 13). Moreover, cryo-SEM images of white cells in *L. debelius* (Supplementary Fig. 13) display a similar ultrastructure to that observed in Fig. 2. This implies that partially ordered, isoxanthopterin particles are widespread in white chromatophores in shrimp. The structures of these 1D ordered isoxanthopterin assemblies are reminiscent of a columnar liquid crystal, where flat and rigid molecules are stacked in cylindrical structures^39^. Notably, liquid crystals are widespread in biology^40,41^, but their importance in biological optics has not been widely investigated. The iridescence of *Pollia* fruit and scarab beetles are known to derive from cellulose and chitin structured like cholesteric liquid crystals, whose helicoidal pitch dictates the colour of the photonic crystal^42–45^. However, the influence of preferred molecular orientation on the properties of scatterers or pigments has not been described.

### Optical properties of individual nanospheres

**Fig. 4:**
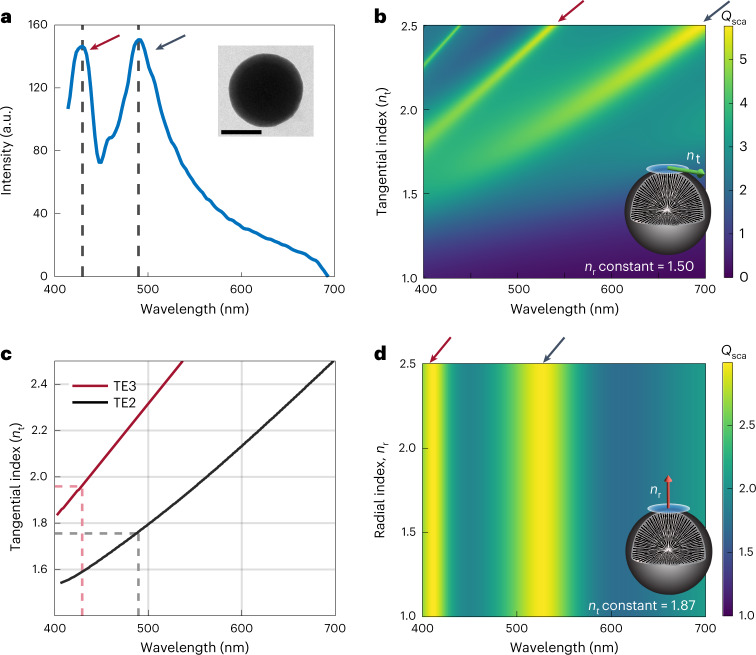
Optical properties of individual isoxanthopterin nanospheres. **a**, Measured dark-field (forward-scattering) spectrum of a single nanosphere. Inset: TEM image of the nanosphere. Scale bar, 200 nm. **b**, Calculated scattering cross-section, *Q*_sca_, as a function of *n*_t_ for a particle with the same dimensions as that in **a** with the radial refractive index, *n*_r_, fixed at *n*_r_ = 1.50. The *Q*_sca_ value is normalized by dividing by the physical cross-sectional area of the particle. **c**, Peak positions of the calculated TE3 and TE2 from **b**, with varying *n*_t_. Dashed lines mark the peak positions of the TE3 and TE2 resonances (1.96 and 1.76, respectively) measured in **a**. **d**, Calculated *Q*_sca_ as a function of *n*_r_ for the same particle, with *n*_t_ fixed as 1.87 (the average tangential index derived from the TE2 and TE3 experimental peak positions). The schematic particles in **b** and **d** show the refractive index varied in each calculation. Red arrows and black arrows at the top indicate the TE3 and TE2 peaks, respectively.

The effect of a preferred molecular orientation on the optics of the nanospheres was investigated using the single-particle scattering method of Beck et al.^35^, as used previously to determine the refractive index of crystalline isoxanthopterin nanospheres. The method enables the tangential refractive index (*n*_t_) of the nanospheres to be deduced from the positions of the Mie resonances in single-particle scattering spectra. Figure 4a shows the forward-scattering spectrum of a single nanosphere and Fig. 4b the calculated scattering cross-section (*Q*_sca_) of such a nanosphere for different tangential indices (with the radial refractive index, *n*_r_, constant). The transverse-electric quadrupole (TE2) and transverse-electric octupole (TE3) resonances are the main contributors to the scattering in the visible (Supplementary Fig. 14). The peak positions of the calculated resonances, which vary with *n*_t_, correlate with the TE3 and TE2 peaks of the experimental spectrum (Fig. 4c). This enables the tangential refractive indices of the nanospheres to be extrapolated from the model, resulting in an average *n*_t_ of 1.87, which is almost identical to that determined for crystalline isoxanthopterin spherulites (*n*_t_ = 1.88)^35^. Due to inhomogeneities in the extracted particles used in these scattering measurements (that is, the nanospheres may contain small voids either intrinsically or due to the extraction process), we anticipate that the actual value of the tangential index of the material is much closer to the DFT-calculated value of 1.96 (ref. ^35^). The results suggest that the origin of the nanospheres’ high refractive index does not lie in the crystalline order, but in the alignment of the highly polarizable plane of isoxanthopterin tangentially to the particle surface (Fig. 3b–f)^46^. As in previous work^35^, calculations of the scattering cross-section (*Q*_sca_) with a fixed *n*_t_ (Fig. 4d) show that the scattering is not affected by the radial refractive index, *n*_r_. The difference between the *n*_t_ values deduced from the TE3 and TE2 peaks is probably due to material dispersion (a higher refractive index is expected for shorter wavelengths, in proximity to the absorption peak of isoxanthopterin in the ultraviolet)^35^. The similarity between *n*_t_ of the 1D ordered spherulites observed here and that of previously observed 3D ordered crystalline ones leads us to conclude that the radial refractive index of the two systems, perpendicular to the molecular stacking plane, is also similar and has a value of *n*_r_ ≈ 1.40. Considering the above discussion and the similarity between the measured and calculated tangential refractive index, we chose to use the DFT-calculated values of the tangential and radial refractive index in the calculations described in the following.

### Optical properties of nanosphere assemblies

**Fig. 5:**
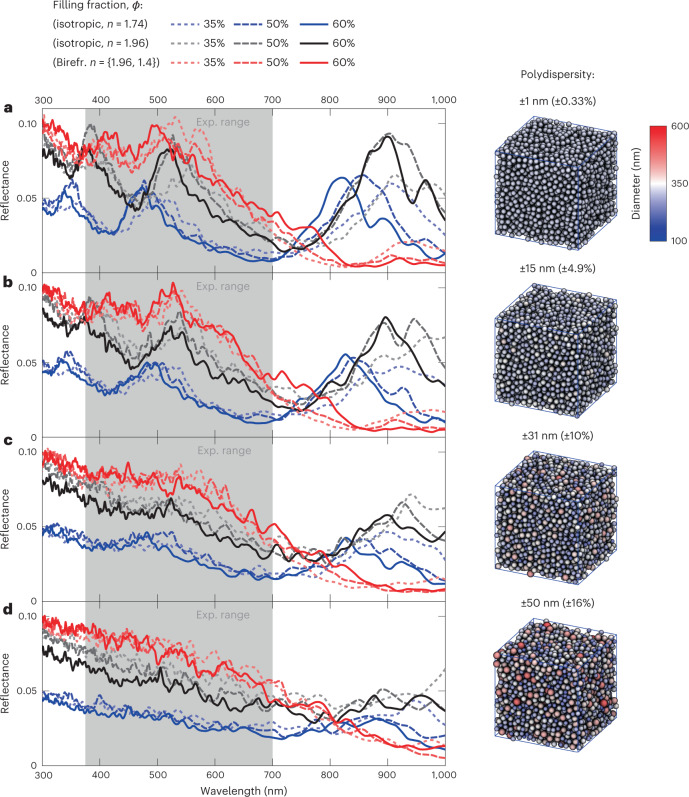
Simulated reflectance of nanosphere assemblies. MD and FDTD simulations of 5 × 5 × 5-μm^3^ photonic glass slabs comprising nanospheres with varying refractive index, polydispersity and filling fraction. Blue trace, isotropic particles (*n* = 1.74); black trace, isotropic particles (*n* = 1.96); red trace, birefringent particles (*n* = 1.40, *n* = 1.96). Filling fractions: 35% (fine dashed trace), 50% (dashed trace), 60% (solid trace). **a**–**d**, Results are shown for polydispersities of 0.33% (**a**), 5% (**b**), 10% (**c**) and 16% (**d**). Absorbing boundary conditions were used in the FDTD simulation. Right: snapshots of the MD-simulated particle configurations with 50% filling fraction. The grey region in the reflectance plots (left) represents the visible range.

Whiteness is a multiple scattering effect which is difficult to achieve with thin layer thicknesses due to the limit on packing density. The optimal filling fraction for scatterers is 20–50% and is strongly dependent on the refractive-index contrast^2,7,47^. Beyond this range, structural correlations emerge, giving rise to colours and/or to a reduction in scattering efficiency due to near-field coupling of the evanescent fields of neighbouring scatterers^6,32^. The unexpected observation of ultra-thin cells containing particle filling fractions of ~50–65% motivated us to explore how whiteness is optimized in these assemblies. We used molecular dynamic (MD) simulations to generate particle assemblies resembling a photonic glass—a disordered assembly of dielectric spheres^48^. Simulations were performed with the experimentally measured particle sizes (305 nm; Supplementary Figs. 15–17 and Supplementary Table 1), with varying polydispersity, filling fractions and layer thicknesses to account for the variation in these parameters in different cells. Reflectance spectra generated from these assemblies were then simulated using the finite-difference time-domain (FDTD) technique. At low polydispersity (<5%), structural correlations give rise to strong reflectance peaks in the infrared and at ~500 nm and 350–400 nm (Fig. 5a,b). On increasing the polydispersity to the experimentally observed 10% (305 ± 31 nm) and above, the structural peaks in the visible are dampened, resulting in broadband reflectance across the visible (Fig. 5c,d).

**Fig. 6:**
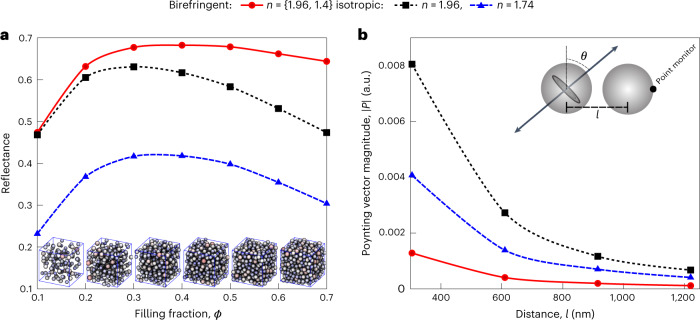
Effect of birefringence on reflectance and near-field coupling. **a**, Total reflectance values for 305-nm isotropic (blue and black traces) and birefringent (red trace) particles under periodic boundary conditions (comparable to a reflectance spectrum obtained using an integrating sphere) as a function of filling fraction. Unlike in the isotropic cases, the birefringent particles show high reflectance even up to 70% filling fractions. Sub-volume snapshots from 5 × 5 × 5-μm^3^ simulation cubes for different filling fractions are shown as insets. **b**, Simulations of coupling between a point source and a point monitor as a function of distance and refractive index for the two-particle (*d* = 305 nm) geometry presented in the inset. Curves present the mean value of two point-source orientations (*θ* = 0° and 90°). See Supplementary Fig. 18 for full data. Birefringence reduces coupling between the particles, leading to increased scattering efficiency in bulk at higher filling fractions, as shown in **a**.

To test the effect of the molecular orientation of the nanospheres on their optical properties, we compared the reflectance of hypothetical isotropic particles with *n* = 1.74 (the average of isoxanthopterin’s three calculated refractive indices; Fig. 5, blue traces) with birefringent particles with *n*_t_ = 1.96, *n*_r_ = 1.40 (the DFT-calculated refractive index for isoxanthopterin^33^; Fig. 5, red traces). The reflectance of the birefringent particle assemblies is double that of the isotropic particles (Fig. 5a–d). Moreover, even at low polydispersity (<5%), the structural peaks of the birefringent assemblies in the infrared and visible are flattened with respect to the isotropic assemblies (Fig. 5a,b). To test whether the enhanced intensity and dampening of the structural peaks was due to birefringence per se or to the elevated refractive index of the birefringent nanospheres, we compared the reflectance of the birefringent particles (*n*_t_ = 1.96, *n*_r_ = 1.40) to isotropic particles with a refractive index of 1.96 (Fig. 5, black trace). Unexpectedly, despite having a lower average refractive index, the birefringent assemblies have a significantly higher reflectance intensity and weaker structural peaks than the isotropic particles (Figs. 5a–d and 6a). This indicates that the anisotropic orientation of refractive indices in the birefringent particles reduces the detrimental effects of near-field coupling, which decreases the scattering efficiency at high filling fractions. To further explore this phenomenon, we plotted the integrated reflectance over the visible range as a function of the filling fraction for the birefringent (*n*_t_ = 1.96, *n*_r_ = 1.40) and isotropic particle assemblies (*n* = 1.96 and *n* = 1.74) (Fig. 6a). This shows that the optimal filling fraction for both isotropic particle assemblies is 30%, with the scattering decreasing above these values, as is typical for particle-based scatterers. In contrast, for the birefringent particles, the intensity is robust against changes in the filling fraction and remains high even up to exceptionally high filling fractions of ~65%, very close to the packing limit of identical spheres. This highlights the unexpected contribution of extreme birefringence to enhanced scattering, providing an additional structural handle through which scattering properties can be controlled. To better understand the apparent reduction of near-field coupling in the birefringent case, we performed additional simulations using the simple geometry of pairs of spheres with a varying centre-to-centre distance, *l*. We placed an emitting dipole in the centre of one sphere and monitored the magnitude of the Poynting vector at the far end of the other sphere (Fig. 6b). Regardless of the distance *l*, the coupling, reflected by the magnitude of the Poynting vector (averaged over the two emitter dipole orientations), is significantly smaller for the birefringent case, even with respect to the lower-index (*n* = 1.74) spheres. We note that this is also true for other dipole emitter positions (Supplementary Fig. 18). The reduced coupling to a near-neighbour particle can be rationalized by scrambling of the polarization by the birefringent medium, inhibiting the strong coupling expected for dipole orientations perpendicular to the line separating the centres of the two spheres.

## Discussion

Our results show that optimization of the scattering of single particles (high refractive index, extreme birefringence) and particle assemblies (polydispersity, high packing density) enables the cleaner shrimp to produce bright whiteness from a device with dimensions of a few micrometres. The scattering from this structure is much more efficient than most artificial photonic glasses, which typically require tens of micrometres of sample to produce high reflectance^49,50^. The thinnest ultra-white structure reported in nature is in the *P. rapae* butterfly, where a reflectance of ~75% is generated by scales <5 μm thick, containing sparsely populated granules of leucopterin^26–28^. However, in contrast to the *P. rapae* butterfly^26–28^ and *Cyphochilus* beetle^20–25^ the bright white colours of the shrimp are generated in the relatively high-refractive-index medium of water, making it more challenging to obtain contrast with the surrounding medium. The selective pressure to generate intense colouration in an aqueous medium presumably underlies the impressive optimization and efficiency of this device. In their natural marine habitat, the white stripes of cleaner shrimp are used as a signal to attract client fish^51,52^, and their intense brightness may provide an advantage in more effective signalling.

At the single-particle level, the high index and birefringence of the nanospheres derives from the radial alignment of stacked, liquid-crystalline isoxanthopterin assemblies. Optical measurements show that the scattering of these particles is dictated by their high tangential refractive index, which arises not from their crystallinity (as documented in previous works on crystalline isoxanthopterin^34,35^), but from the preferred orientation of isoxanthopterin molecules. The 1D stacking arrangement seen here is reminiscent of biogenic melanin, which displays a single broad stacking peak in X-ray scattering measurements^53,54^. Although the role of liquid crystallinity and molecular orientation has previously been shown in photonic crystals^42–45^ and here in a scattering system, its influence on the optical properties of absorptive pigments is surprisingly not well established.

At the level of the particle assemblies, FDTD simulations show that a polydispersity of 10% dampens structural peaks, preventing the emergence of colours. The organisms’ subtle control over these structural parameters is illustrated by comparison with the tapetum of crustacean eyes^34^. In the tapetum, blue colours are produced by assemblies of crystalline isoxanthopterin nanospheres with only a 5% lower polydispersity. Most intriguingly, the anisotropic distribution of refractive indices in the birefringent particles mitigates against the effects of near-field coupling and optical crowding observed in isotropic spheres of the same index. This phenomenon is key to obtaining a dense particle packing, which facilitates the ability to generate whiteness from ultra-thin layers. The present study on the effects of birefringence on scattering from disordered assemblies, combined with past work on ordered birefringent assemblies (where birefringence results in partial stop bands in the band diagram of an opal^36^), strengthens our understanding that systems harbouring extremely birefringent (~30%) media are, in many respects, fundamentally different from optically isotropic ones.

## Conclusions

This work elucidates the structural and optical strategies utilized in the cleaner shrimp to generate one of biology’s most efficient white materials. Aside from its implications for the fundamentals of light-scattering physics, the results provide inspiration for the design of organic, biologically benign replacements for artificial scatterers like TiO_2_, which are in urgent demand. We show that, by controlling the orientation and assembly of biological nutrient molecules with high polarizabilities, optical structures may be generated with comparable or superior performance to analogous artificial devices. Finally, this work highlights how the extreme birefringence obtainable in crystals (or liquid crystals) of small organic molecules (~30%) can compensate for the typically lower indices of these materials relative to inorganics and serve as a basis for optimal scattering layers.

## Methods

### Specimen collection

Adult *L. amboienesis* were purchased at a local pet shop in Beer-Sheva, Israel. Use of crustaceans for research purposes does not require animal ethics approval in Israel, but animals were handled with established best practices.

### Optical microscopy

A Zeiss Discovery.V20 stereomicroscope equipped with an Axiocam 305 colour camera was used to take low-magnification images of the cleaner shrimp. High-magnification optical microscopy images were taken using a Zeiss AX10 microscope equipped with a Zeiss Axiocam 705 colour camera using epi-illumination and imaging in reflection mode with ×2.5, ×5, ×10, ×20 and ×50 air objectives.

### Microspectrophotometry

Reflectance spectra were obtained from the white stripe and front maxillipeds of the cleaner shrimp using a Zeiss AX10 microscope equipped with a Zeiss ×10/0.25 NA HD DIC objective and a Zeiss C-Apochromat Water ×40/1.20 NA objective. The specimens were epi-illuminated with an LEJ HXP 120-V compact light source. The reflected light was collected from the same objective into an optical fibre (QR450-7-XSR, Ocean Insight) and the spectra measured using an Ocean Insight FLAME miniature spectrometer. The spectra were normalized using a white diffuse reflectance standard (Labsphere USRS-99-010, AS-01158-060). Background spectra were measured when no light was applied. Reflectance spectra were obtained over the range of 180–880 nm. The integration time ranged from 10 to 40 s, with 10–20 averaged scans for each measurement.

### Cryogenic-SEM

White maxillipeds of the cleaner shrimp (not chemically fixed) were cut perpendicular to the length of the limb into ~1-mm sections. The sections were sandwiched between two aluminium discs and cryo-immobilized in a high-pressure freezing device (EM ICE, Leica). The frozen samples were then mounted on a holder under liquid nitrogen in a specialized loading station (EM VCM, Leica) and transferred under cryogenic conditions (EM VCT500, Leica) to a sample preparation freeze-fracture device (EM ACE900, Leica). The samples were freeze-fractured at −120 °C, etched at −110 °C for 3 min, and coated with 3 nm of Pt/C. The samples were imaged in an HRSEM Gemini 300 scanning electron microscope (Zeiss) by a secondary electron in-lens detector while maintaining an operating temperature of −120 °C.

### SEM

A piece of fresh tissue dissected from *L. amboienesis* dorsal white stripe was glued to an SEM stub using conductive carbon adhesive tape and allowed to dry at room temperature. The sample was coated with 5-nm iridium (Quorum Technologies, Q150T). Imaging was performed using a Helios G4 UC dual-beam field-emission scanning electron microscope.

### Raman spectroscopy

A cleaner shrimp (*L. amboienesis*) was euthanized before the experiment by placing in ice for ~10 min. The abdomen was placed on a glass slide covered with aluminium foil. Raman spectra were obtained from the white stripe on the cleaner shrimp’s abdomen. Synthetic isoxanthopterin was purchased from Sigma Aldrich. Micro-Raman measurements were performed with a confocal Horiba LabRam HR Evolution system, equipped with a Syncerity charge-coupled device (CCD) detector (deep-cooled to −60 °C, 1,024 × 256 pixels). Lasers (633-nm and 785-nm) were used as excitation sources, and the power on the sample was 12 mW and 30 mW, respectively. The laser was focused with a ×50 LWD objective (Olympus LMPlanFL-N, NA 0.5), ~50 μm deeper than the surface of the shrimp abdomen so that the focal point was under the cuticle. The measurements were taken using a 600-g-mm^−1^ grating and a 100-μm confocal pinhole. The typical exposure time varied from 10 to 180 s. The spectra were collected and baselined using LabSpec software (version 6.5.1.24).

### Ultra-thin tissue sectioning for TEM imaging

A chemically fixed piece of antenna was washed with 0.1 M cacodylate buffer for 2 h and post-fixed with 2% osmium tetroxide for 2 h. The sample was washed with 0.1 M cacodylate buffer three times for 5 min and two times with double distilled water (DDW). The sample was immersed in 1% tannic acid for 2 h, then washed three times in DDW. The sample was incubated with 2% uranyl acetate in DDW for 1 h in the dark, and washed with DDW three times for 5 min. The sample was dehydrated by washing in an ethanol series (30%, 50%, 70%, 90% and 100% ethanol vol/vol). At each ethanol concentration, the sample was washed twice for 10 min. The sample was further dehydrated with acetone three times for 5 min each. For the embedding, the sample was kept overnight in a solution of 50% EMbed 812 (EMS #14120) with acetone. Later, the solution was replaced by 100% EMbed 812 and left for 2 h. After 2 h, the 100% EMbed 812 was replaced and left for a further 2 h. In total, washing with 100% EMbed 812 was performed three times. The final EMbed 812 suspension was left overnight. Final embedding in a mould was carried out at 60 °C for 48 h. Ultra-thin sections were prepared with an ultra-microtome (RMC) and imaged with a Tecnai T12 TWIN TEM (FEI) and HRSEM Gemini 300 SEM (Zeiss) STEM detector in dark-field (DF) mode.

### TEM imaging and electron diffraction

The nanospheres were extracted from the white maxillipeds and antennae of a cleaner shrimp (Supplementary Fig. 15). To do this, the maxillipeds and antennae were sliced into ~1–2-mm pieces, placed in a glass vial with a few drops of hexane, and sonicated for 30 s in a sonication bath, then 3 µl of the resulting suspension was dropped on a carbon-coated Cu-meshed TEM grid and allowed to dry. The resulting samples were observed with a ThermoFisher Scientific (FEI) Tecnai T12 G2 TWIN TEM operating at 120 kV. Images and electron-diffraction patterns were recorded using a Gatan 794 MultiScan CCD camera. Electron-diffraction analysis was done using Gatan DigitalMicrograph software with the DIFPack module. Particle diameters were measured using Gatan DigitalMicrograph software, and 146 particles were measured to find the average size and standard deviation (Supplementary Table 1 and Supplementary Figs. 16 and 17).

### Synchrotron radiation in situ μ-spot wide-angle X-ray diffraction

A 0.5-cm piece of a white maxilliped (not chemically fixed) was sealed inside X-ray transparent foil and placed on a sample holder. The measurements were performed at the PXI-X06SA beamline at the Swiss Light Source. The wavelength of the beam was 0.9999 Å, and the diffraction patterns were measured by an EIGER 4M detector at a sample–detector distance of 130 mm or 200 mm. The beam size was 10 μm × 10 μm. The exposure time was 0.5 s. The radial integration of the 2D scattering patterns was analysed using ALBULA and dpdak software packages.

### Refractive-index measurements

The sample was prepared as described for TEM imaging, then drop-cast on a carbon-coated Cu-meshed reference grid. Front scattering measurements were performed using a commercial optical microscope (Zeiss, Axio Observer 5). The sample was illuminated with a ‘white’ light-emitting diode in a dark-field configuration and collected using an objective (N-Achroplan, ×20, NA 0.45). The image was directed outside the microscope into a custom set-up that further magnified the image (×6) and coupled into a fibre (105-µm in diameter) and then into a spectrometer (Shamrock 303i, Andor). The particles were illuminated using an annulus (*Θ*_i_ = 25°) that partially entered the objective. Unscattered light was filtered out using an iris placed at the Fourier plane (imaged by a Blackfly S USB3, FLIR). The diameter of the nanospheres was extracted using DigitalMicrograph (GATAN). Calculations of the scattering cross-section were done using MATLAB, as described for the Mie coefficients in the following section. Detailed information about the calculations of the scattering cross-section is available in ref. ^35^.

### Simulated reflectance spectra

MD simulations were carried out using HOOMD-blue (version 2.9.4)^57^, with the Langevin integrator (*kT* = 0.05) and polydisperse 12–0 soft-core pair-potential:

$${\sigma \left( r \right)} = \left\{\begin{array}{ll} {}^{v_{0}} \left[\left(\frac{\sigma_{ij}}{r}\right)^{12} - \left(\frac{\sigma_{ij}}{r} \right)^{0} \right] + \sum\nolimits_{0}^2 C_{k}\left(\frac{r}{\sigma_{ij}} \right)^{2k}, \,{r/\sigma_{ij} \le r_{\rm{c}}}\\0 & {\rm{otherwise}} \end{array} \right.$$

$${\sigma _{ij}} = {\frac{1}{2}\left( {\sigma _i + \sigma _j} \right)\left( {1 - {\it{\epsilon }}\left| {\sigma _i - \sigma _j} \right|} \right)}$$

where *v*_0_ = 1, *ϵ*_*ij*_ = 0.01, *σ*_*a*_ is the diameter of particle *a*, and *r*_c_ = 1.5*μ*_1_ is the cutoff distance, using the PolydisperseMD plugin^58^. Particle sizes were randomized from a log-normal distribution, with mean ${\mu _2} = {\log \left( {\mu _1^2/\sqrt {\mu _1^2 + s_1^2} } \right)}$ and ${\sigma _2^2} = {\log(1+{\sigma_1^2}/{\mu^2_1})}$, and the values’ outside range [*d*_min_, *d*_max_] was rounded to the nearest boundary to avoid the chance of extreme particle sizes. In the simulations, mean particle size *μ*_1_ = 305 nm, *σ*_1_ = {1, 15, 31, 50 nm}, *d*_min_ = 100 nm, *d*_max_ = 600 nm. Simulations were initiated with a simple cubic lattice in a 20 × 20 × 20-μm^3^ box with periodic boundary conditions, which was then squeezed to a final 5 × 5 × 5-μm^3^ volume during the rapid quench, in 5 × 10^5^ time steps. Particle coordinates and diameters were then imported to Lumerical FDTD (Ansys). In the FDTD simulation set-up, perfectly matched layer (PML) boundaries in the *x* and *y* directions were used (except for Fig. 6a, where periodic boundary conditions (PBC) were used to obtain reflectance values comparable to an integrating sphere measurement), and a plane-wave source and reflection monitor were placed 0.5 and 4.5 μm above the sample (in the *z* direction), respectively, to mimic the numerical aperture of the objective used to collect reflected light in the experimental conditions. The recorded reflectance was integrated in Lumerical over the 5 × 5-μm^2^ monitor area, and in the case of Fig. 6a was spectrally averaged. The two particle-coupling investigations (Fig. 6b) were carried out in Lumerical by placing two particles (*d* = 305 nm) at 1×, 2×, 3× and 4×*d* apart from each other (centre-to-centre distance) with PML conditions, and a point monitor was placed at the far edge of the second particle while the role of the point-source location and orientation was investigated by placing a dipole source at the centre and separately at the edge of the first particle, and the source orientation was independently set either parallel or orthogonal to the axis along the two particles (Supplementary Fig. 18). In all FDTD simulations, the medium refractive index was set to *n* = 1.33. the isotropic particle refractive index was set to either *n* = 1.74 or *n* = 1.96 and, for the birefringent particles, the tangential and radial refractive indices were set to *n* = 1.96 and *n* = 1.4, respectively. MD visualizations were done using VMD^59,60^.

## Online content

Any methods, additional references, Nature Portfolio reporting summaries, source data, extended data, supplementary information, acknowledgements, peer review information; details of author contributions and competing interests; and statements of data and code availability are available at 10.1038/s41566-023-01182-4.

## Supplementary information

Supplementary Information Supplementary Figs. 1–18, Table 1 and references 1–5.

## Data Availability

All data are available in the main text, the Supplementary Information or from ref. ^61^.

## Code availability

All code is available in ref. ^61^.
