# Supplementary material for: Brilliant whiteness in shrimp from ultra-thin layers of birefringent nanospheres
Source: Nat Photonics. 2023 Apr 24;17(6):485–93. doi: 10.1038/s41566-023-01182-4 (PMC10241642; doi:10.1038/s41566-023-01182-4)
Supplement: Supplementary file 1 — Supplementary Figs. 1–18, Table 1 and references 1–5. [file 41566_2023_1182_MOESM1_ESM.pdf]

nature photonics

Article https://doi.org/10.1038/s41566-023-01182-4

Brilliant whiteness in shrimp from ultra-thin layers of birefringent nanospheres

In the format provided by the authors and unedited

Supplementary Information

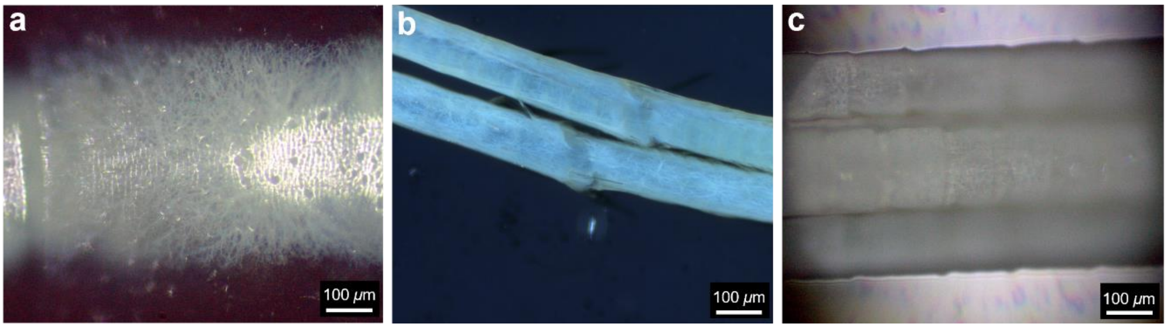

Fig. S1 | Optical micrographs of the white stripe(**a**), white maxillipeds(**b**), and antennae (**c**) of *L. amboinensis* exhibiting increasing degree of interdigitation of the white chromatophores in the tissues.

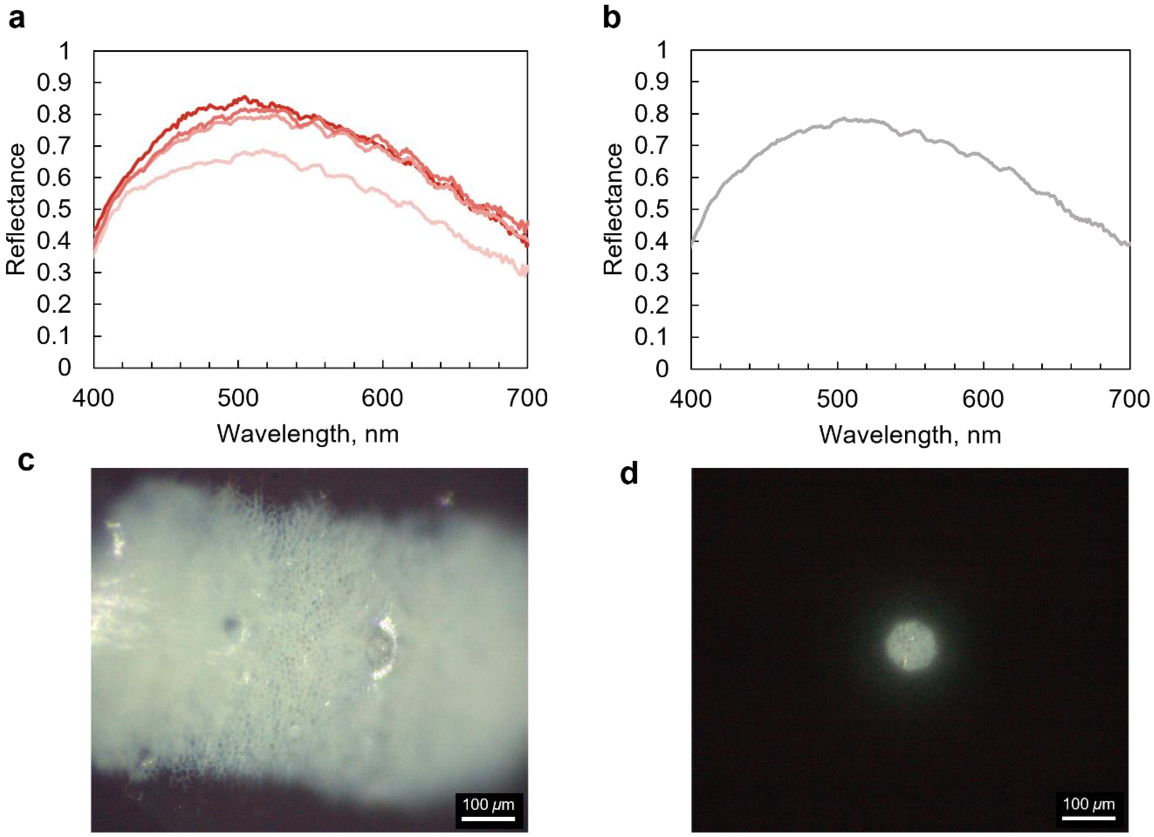

Fig. S2 | a, Reflectivity spectra from four different areas on the dorsal stripe of *L. amboinensis* using a 10X air objective with a small aperture. **b,** Average of the spectra in (**a**). **c,** Optical micrograph of an area on the dorsal stripe before the aperture was closed. **d,** Optical image of the same area as in (**c**), with small aperture, i.e., the area from where the spectrum was obtained.

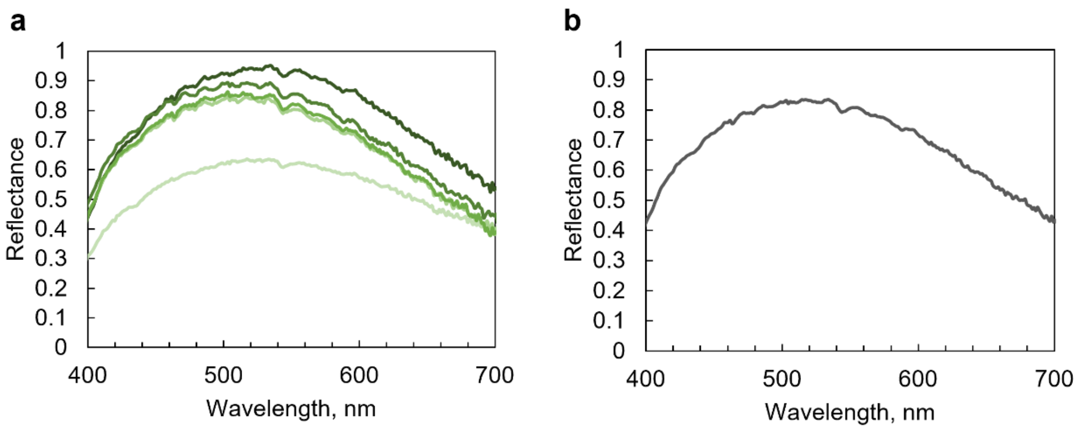

Fig. S3 | a, Reflectivity measurements from five different areas on the dorsal stripe using a 40X water immersion objective with a small aperture. **b,** Average of the spectra in (**a**).

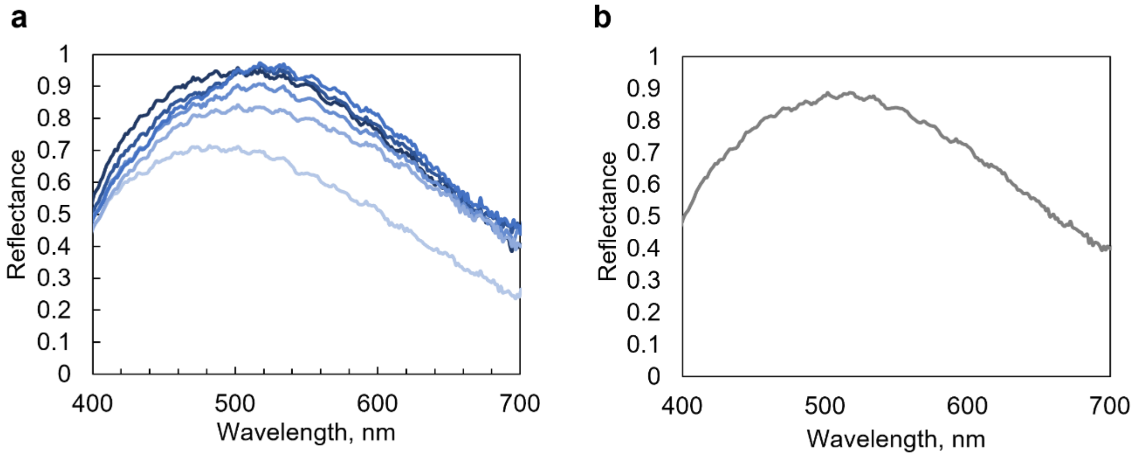

Fig. S4 | a, Reflectivity measurements from six different areas on the dorsal stripe using a 40X water immersion objective with a medium aperture. **b,** Average of the spectra in a.

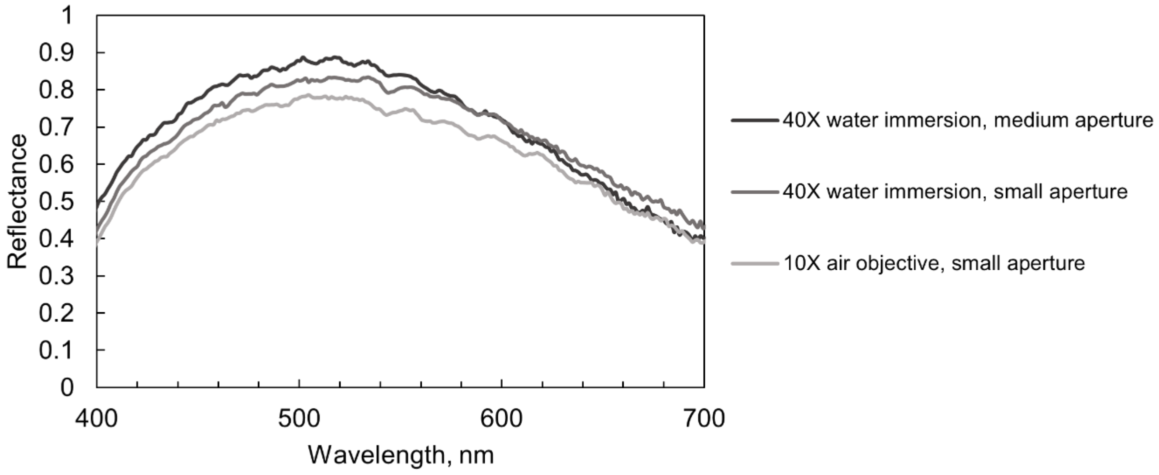

Fig. S5 | Comparison between the average reflectivity spectra obtained with three different microscope conditions exhibiting similar reflectivity response.

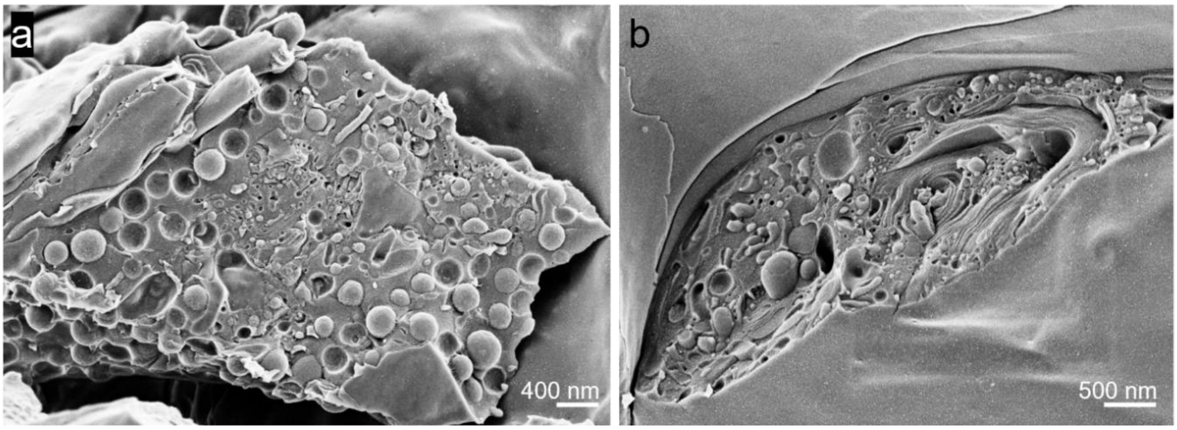

Fig. S6 | a-b, Cryo-SEM micrographs of chromatophore cells containing nanospheres in the white maxilliped of the Pacific Cleaner Shrimp, *L. amboinensis*. The cells contain numerous organelles including features reminiscent of the Golgi apparatus and endoplasmic reticulum (ER).

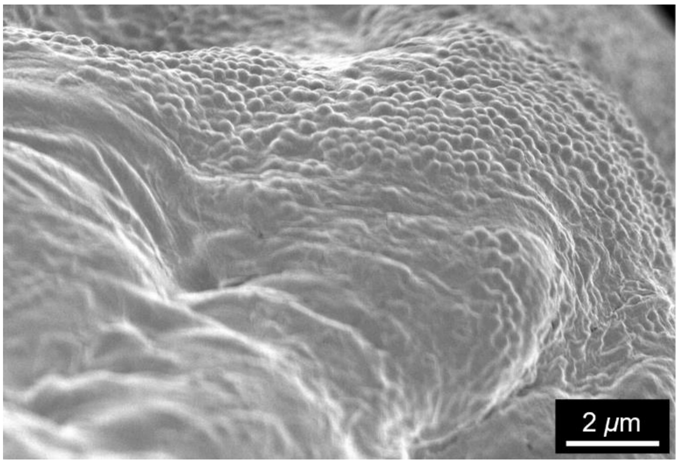

Fig. S7 | SEM micrograph of nanospheres in a tissue extracted from the white stripe of the Pacific Cleaner Shrimp.

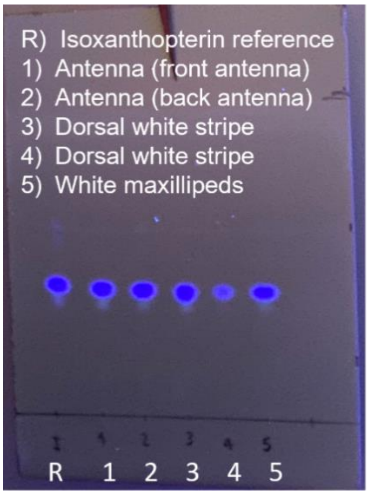

Fig. S8 | Thin Layer Chromatography (TLC) of samples collected from different areas of the Pacific Cleaner Shrimp compared to synthetic isoxanthopterin (Sigma Aldrich). Visualized using a UVA lamp. Solvents: 10% NH_4OH: isopropanol, 1:3.

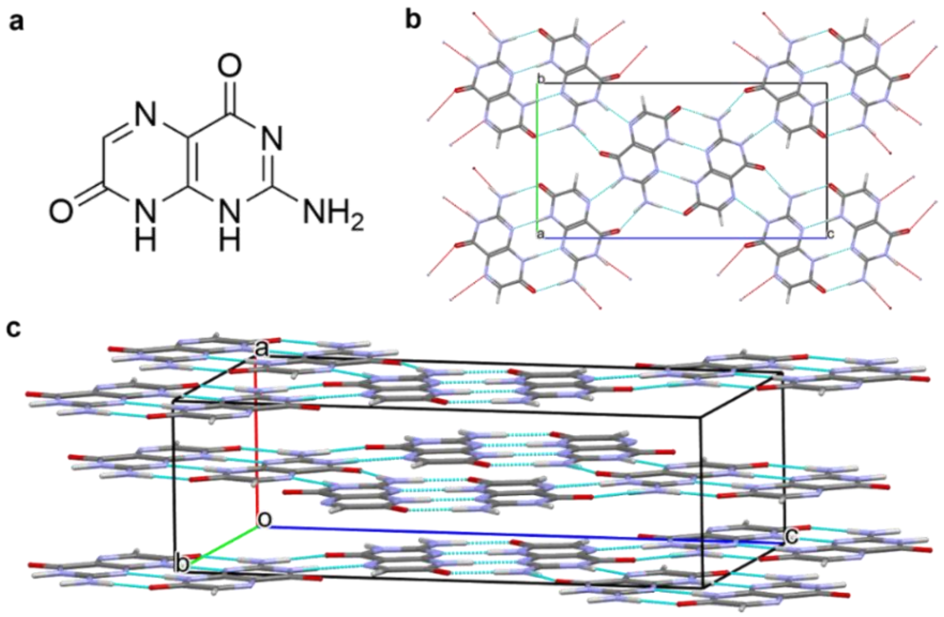

Fig. S9 | a, Molecular structure of isoxanthopterin. **b-c,** The calculated crystal structure of isoxanthopterin viewed perpendicular (**b**) and parallel (**c**) to the H-bonded layer (*bc* plane) of isoxanthopterin molecules (reproduced from ref.[33]).

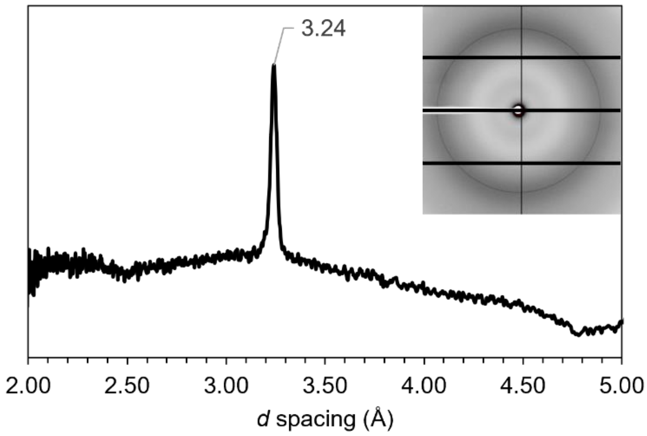

Fig. S10 | *In situ* μ-spot wide-angle X-ray scattering (WAXS) peak obtained by radial integration of the 2D scattering pattern (inset). Obtained from a chemically fixed section of the white stripe of *L. amboinensis*.

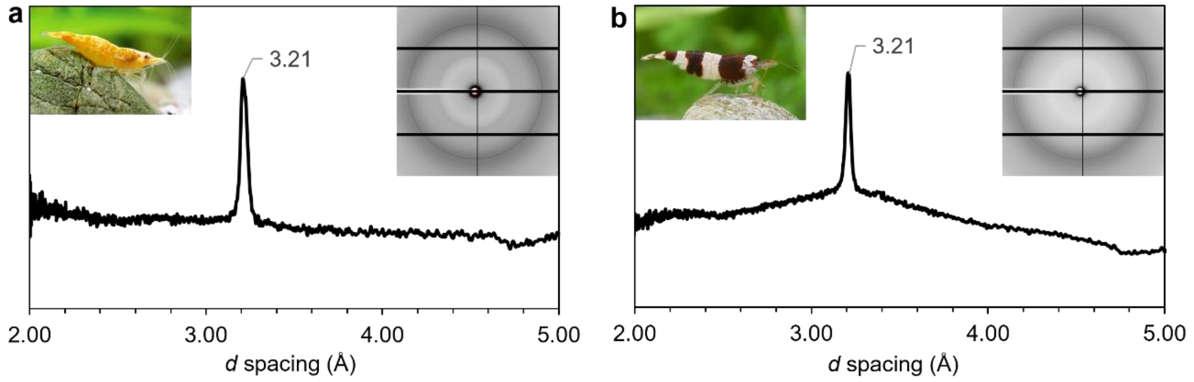

Fig. S11 | *In situ* μ-spot wide-angle X-ray scattering (WAXS) peak obtained by radial integration of the 2D scattering pattern (inset, right) from **a,** white regions of *Neocaridina davidi* (inset, top left) and **b,** white regions of *Caridina breviate* (inset, top left).

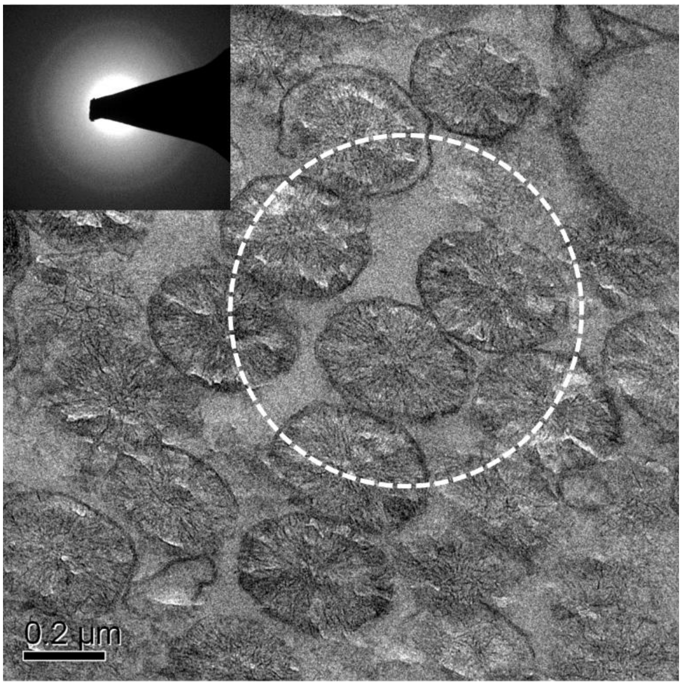

Fig. S12 | TEM micrograph of isoxanthopterin nanospheres in an ultrathin tissue section of an antenna of *L. amboinensis*. Inset; Selected Area Electron Diffraction peak with *d* spacing ~3.2 Å.

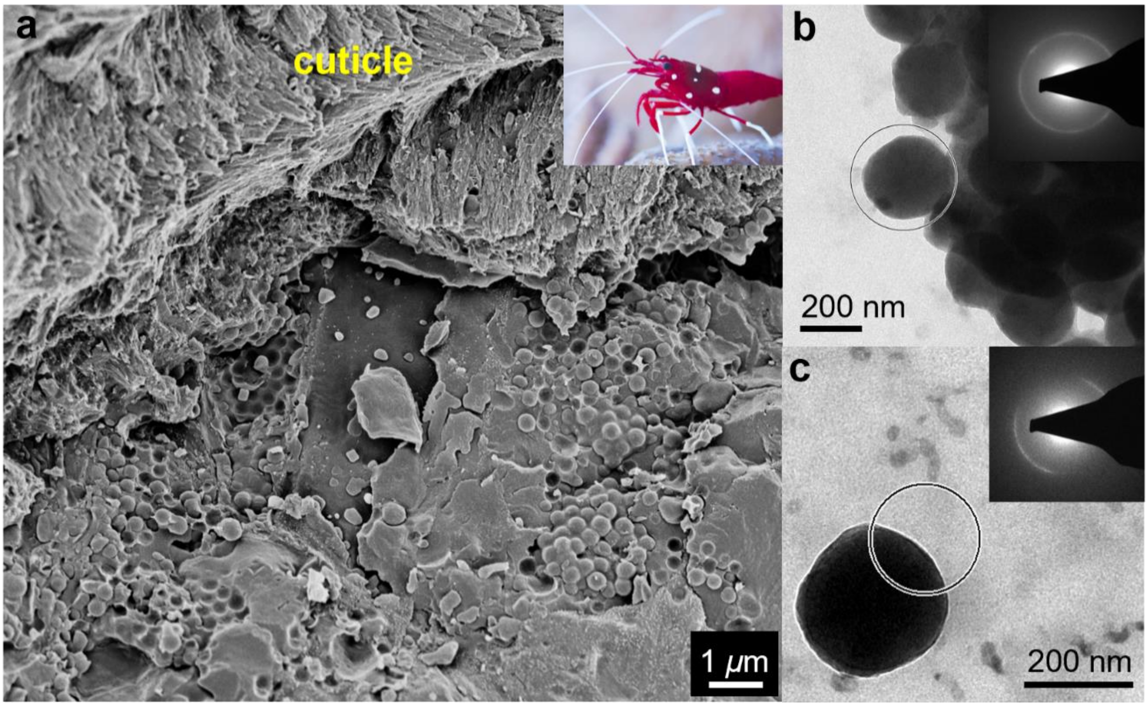

Fig. S13 | a, Cryo-SEM micrograph from the white leg of *Lysmata Debelius* (inset). **b-c,** TEM images and electron diffraction of extracted nanospheres. The nanospheres are very similar in size and appearance to those in *L. amboinensis*, display the same electron diffraction and are contained in cells under the cuticle.

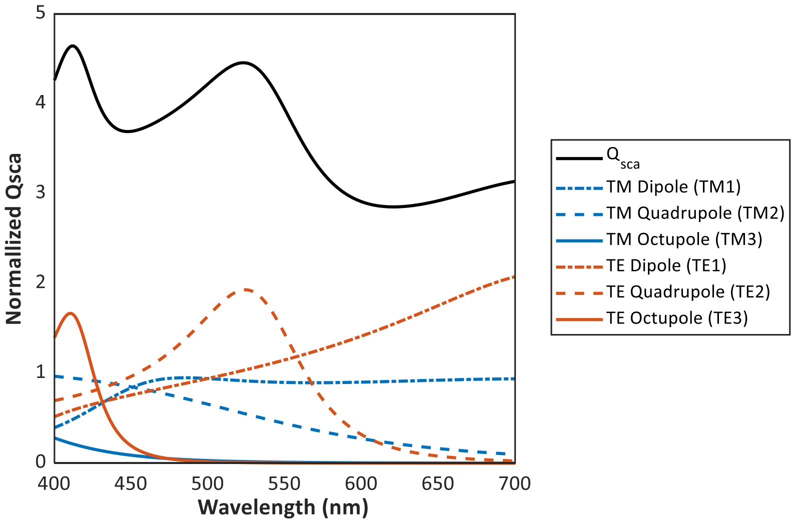

Fig. S14 | Calculated scattering cross-section Q_{sca} (normalized) as a sum of the contribution of each multipole, with the following parameters: particle diameter = 378 nm, n_t=1.87, n_r=1.5, n_{medium}=1, n_{core}=1, t/r=0.999 (effectively all shell). It shows that the main contributors to the Q_{sca} are TE2 and TE3 modes.

Table S1. Particle average size and standard deviation measured from TEM images.

N	Average (nm)	Std (nm)	Min (nm)	Max (nm)
146	305	31	238	407

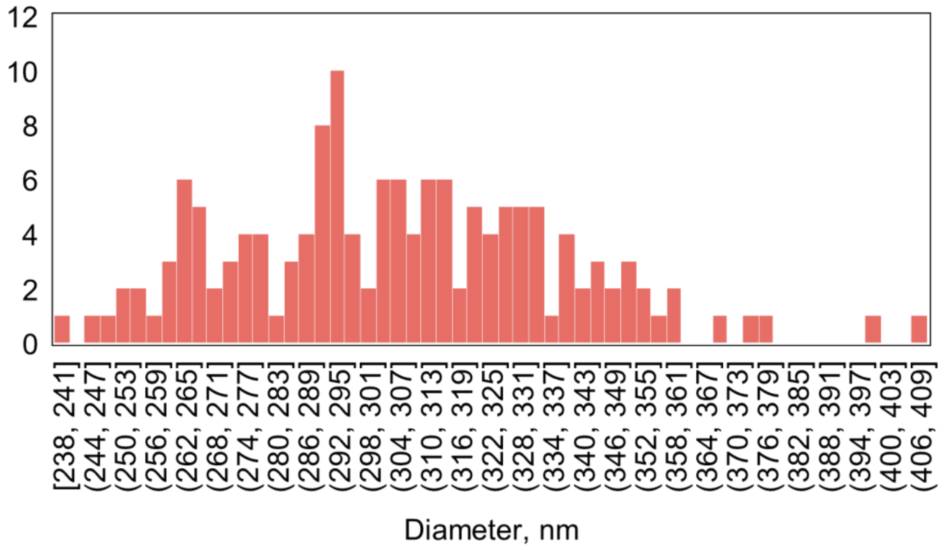

Fig. S15 | Histogram exhibiting the nanosphere size distribution. N(number of particles) = 146, bin size = 3, number of bins: 57.

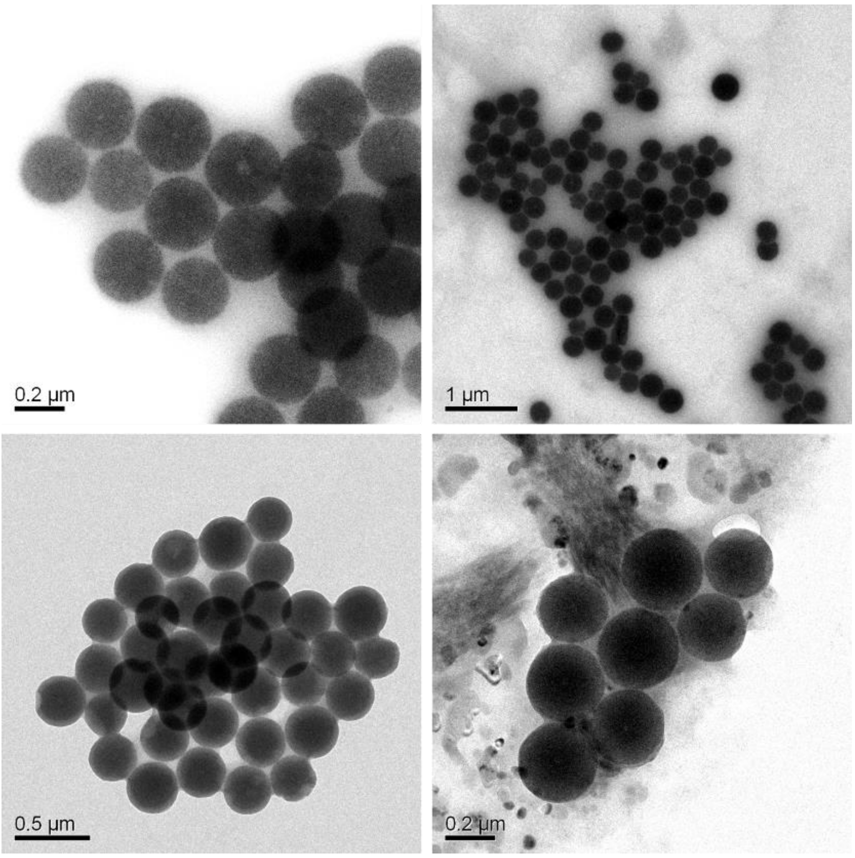

Fig. S16 | Representative TEM micrographs of extracted isoxanthopterin nanospheres from the Pacific Cleaner Shrimp (*Lysmata amboinensis*) used to measure the average diameter and standard deviation of the particles.

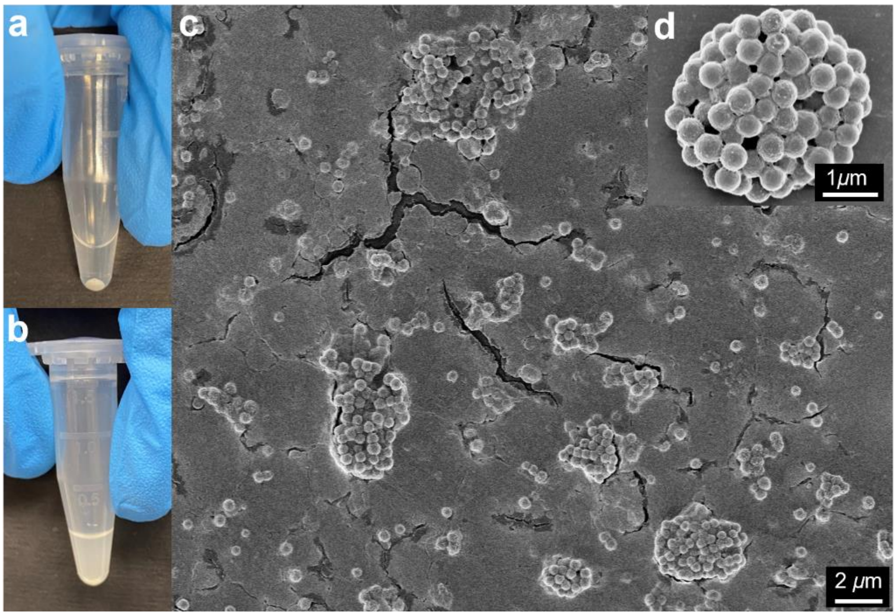

Fig. S17 | a, Extracted nanospheres in a pellet at the bottom of an Eppendorf tube exhibiting white color. **b,** The same sample as in (**a**) with the nanospheres dispersed in the solvent, forming an opaque suspension. **c,** SEM micrograph of the sample in (**a**) and (**b**). **d,** Higher magnification SEM micrograph of the nanospheres from the sample in (**a**) and (**b**).

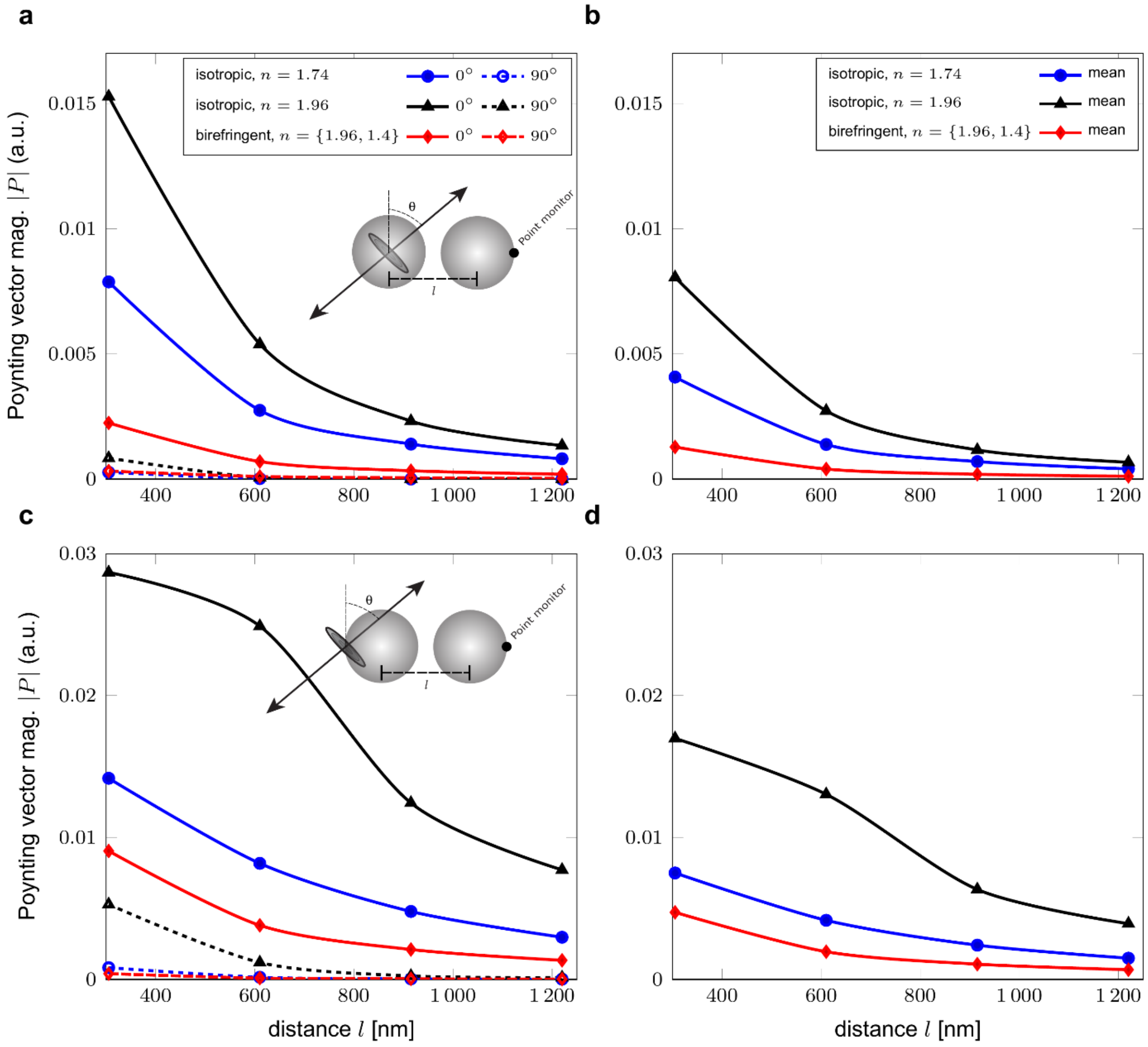

Fig. S18 | Simulations of near-field coupling in a pair of nanospheres as a function of particle-to-particle distance *l* and refractive index distribution *n*. On the left side **a,c,** the recorded intensity ($\propto |P|$) is shown for different point source orientations $\theta = 0°$ and $90°$, and on the right hand side **b,d,** the mean value between the two orientations is shown. In the top row the point source is at the centre of 1st particle, and at the bottom row, on edge of the particle. As can be seen, regardless of position and dipole orientation, birefringence decreases the recorded intensity due to decreased coupling between the two particles.
